# Developing a Machine Learning Algorithm to Predict the Probability of Medical Staff Work Mode Using Human-Smartphone Interaction Patterns: Algorithm Development and Validation Study

**DOI:** 10.2196/48834

**Published:** 2023-12-29

**Authors:** Hung-Hsun Chen, Henry Horng-Shing Lu, Wei-Hung Weng, Yu-Hsuan Lin

**Affiliations:** 1 Department of Mathematics Fu Jen Catholic University New Taipei City Taiwan; 2 Program of Artificial Intelligence & Information Security Fu-Jen Catholic University New Taipei City Taiwan; 3 Institute of Statistics National Yang Ming Chiao Tung University Hsinchu Taiwan; 4 Department of Statistics and Data Science Cornell University Ithaca, NY United States; 5 Computer Science and Artificial Intelligence Laboratory Massachusetts Institute of Technology Cambridge, MN United States; 6 Institute of Population Health Sciences National Health Research Institutes Miaoli County Taiwan; 7 Department of Psychiatry National Taiwan University Hospital Taipei Taiwan; 8 Department of Psychiatry College of Medicine National Taiwan University Taipei Taiwan

**Keywords:** human-smartphone interaction, digital phenotyping, work hours, machine learning, deep learning, probability in work mode, one-dimensional convolutional neural network, extreme gradient-boosted trees

## Abstract

**Background:**

Traditional methods for investigating work hours rely on an employee’s physical presence at the worksite. However, accurately identifying break times at the worksite and distinguishing remote work outside the worksite poses challenges in work hour estimations. Machine learning has the potential to differentiate between human-smartphone interactions at work and off work.

**Objective:**

In this study, we aimed to develop a novel approach called “probability in work mode,” which leverages human-smartphone interaction patterns and corresponding GPS location data to estimate work hours.

**Methods:**

To capture human-smartphone interactions and GPS locations, we used the “Staff Hours” app, developed by our team, to passively and continuously record participants’ screen events, including timestamps of notifications, screen on or off occurrences, and app usage patterns. Extreme gradient boosted trees were used to transform these interaction patterns into a probability, while 1-dimensional convolutional neural networks generated successive probabilities based on previous sequence probabilities. The resulting probability in work mode allowed us to discern periods of office work, off-work, breaks at the worksite, and remote work.

**Results:**

Our study included 121 participants, contributing to a total of 5503 person-days (person-days represent the cumulative number of days across all participants on which data were collected and analyzed). The developed machine learning model exhibited an average prediction performance, measured by the area under the receiver operating characteristic curve, of 0.915 (SD 0.064). Work hours estimated using the probability in work mode (higher than 0.5) were significantly longer (mean 11.2, SD 2.8 hours per day) than the GPS-defined counterparts (mean 10.2, SD 2.3 hours per day; *P*<.001). This discrepancy was attributed to the higher remote work time of 111.6 (SD 106.4) minutes compared to the break time of 54.7 (SD 74.5) minutes.

**Conclusions:**

Our novel approach, the probability in work mode, harnessed human-smartphone interaction patterns and machine learning models to enhance the precision and accuracy of work hour investigation. By integrating human-smartphone interactions and GPS data, our method provides valuable insights into work patterns, including remote work and breaks, offering potential applications in optimizing work productivity and well-being.

## Introduction

The work hours of office workers, including medical staff members, are typically assessed based on the time spent at the worksite. However, with the widespread adoption of remote work during the COVID-19 pandemic, flexible work schedules have become more prevalent. This shift has made it challenging to distinguish break times at the worksite from remote work hours, leading to increased uncertainty in estimating overall work hours. While self-reports have been commonly used to study break times, remote work, and work hours [1-3], they experience potential biases and are not ideal for monitoring longitudinal time periods accurately. Recall biases, in particular, can reduce the reliability of self-reported work hours. Furthermore, distinguishing between being at work (“on-working”) and off work (“off-working”) is not always straightforward. Instead, it involves a spectrum of work-related behaviors, including work performance, work efficiency, and work fatigue.

To address these challenges, artificial intelligence technologies, such as machine learning and deep learning, have been used to classify subjects with high complexity. However, many applications of supervised machine learning require large amounts of manually labeled training data, which is resource intensive and can be subject to biases or poor interrater reliability, especially in behavioral science. For individuals who primarily work in 1 specific location, defining their working state is relatively simple. In this context, we developed an app called “Staff Hours,” which uses GPS background data to automatically calculate users’ work hours. The app also records users’ screen events, such as notification timestamps, screen on or off events, and types of apps used. These screen events have been shown to be informative in various aspects of human behavior [4-9]. Given the ubiquity of smartphones in modern work styles, measuring long-term human-smartphone interactions through mobile apps not only reflects cognitive functioning in real time [10] but also provides behavioral insights that may differ between on-working and off-working states. Specifically, the timestamps of notifications, screen on or off events, and types of apps used correspond to stimuli, individual responses, and the content of stimuli or responses in mental chronometry [11]. One essential aspect of cognitive ability is reaction time, which is typically measured by the elapsed time between a stimulus onset and an individual’s response in a laboratory setting. In a naturalistic setting, continuous reaction time measurements can be obtained through the elapsed time between notification onset and the smartphone user’s response with the screen on. These continuous reaction time measurements via human-smartphone interactions may serve as an indicator of work mode [12].

In this study, we used data sets of human-smartphone interaction patterns and GPS-defined locations to develop a machine learning–based indicator of work mode probability. It was hypothesized medical staff members exhibit distinct human-smartphone interaction patterns between on-working and off-working states. Our specific aims were to (1) evaluate the accuracy of the machine learning algorithm in distinguishing human-smartphone interaction patterns between GPS-defined on-working and off-working periods and (2) identify the distribution and proportion of break times during GPS-defined work hours and remote work hours during GPS-defined off-working periods based on the probability of being in work mode.

## Methods

### Study Sample

We collected data from 121 medical staff members who had at least 5 days’ worth of data representing their “typical work hours” between May 2018 and April 2022. The data were obtained from the Staff Hours database owned by the National Health Research Institutes. The Staff Hours app, used in the study, automatically estimated users’ work hours on a daily basis through GPS records using a previously described algorithm [4,13,14]. All participants volunteered for the study and had installed the app, providing informed consent for electronic data collection. The app was available on both Android and iOS platforms.

The typical work schedule considered in the study had the following characteristics: (1) off-working status at the beginning and end of the day (excluding night shifts and on-call duties), (2) on-working status starting before noon, and (3) work duration longer than four hours. Data from each participant were included for analysis if they met these criteria for at least 5 days. This 5-day threshold was chosen based on the common 5-day workweek schedule of modern workplaces, and it was deemed sufficient to capture the range of human-smartphone interaction patterns at work. The machine learning models were trained on the selected typical work schedule, and data were analyzed over holidays, defined as days without records of GPS location at the worksite (indicating 0 work hours).

### Ethical Considerations

The study was approved by the institutional review board of the National Health Research Institutes (EC1100109-E), and all clinical investigations were conducted in accordance with the Declaration of Helsinki.

### Design of the Staff Hours App

**Figure 1:**
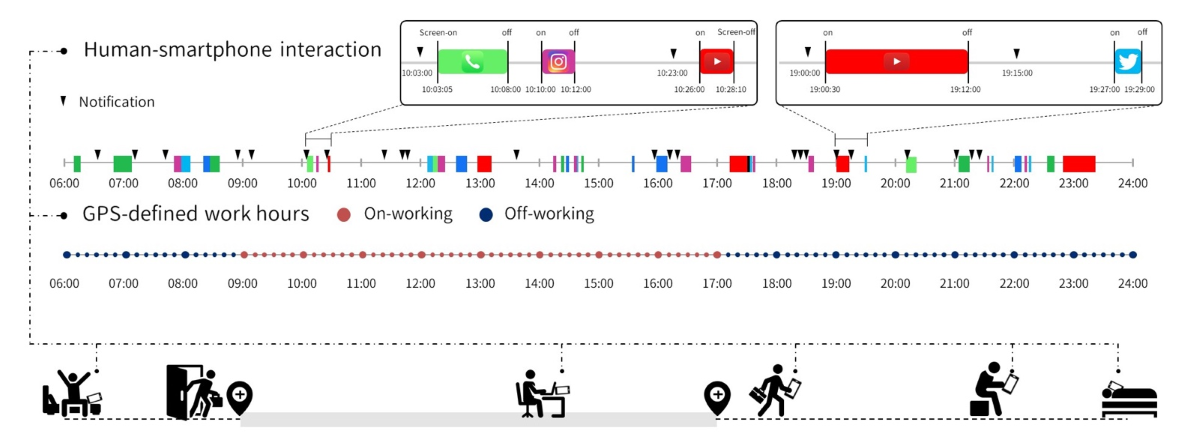
The measurement of human-smartphone interaction patterns and GPS locations in this study. The Staff Hours app simultaneously recorded users’ GPS location and smartphone events as 3 key variables: timestamps of the screen on or off events, notifications, and labels of the app in use. The boxes at the top-right corner show the details of the smartphone events. The temporal resolution of screen events was 1 second. The sampling rate of GPS data was fixed at 10 minutes. GPS-defined work hours started when the workplace location was within range for a consecutive 1800 seconds and ended when there was a consecutive 1800-second period without the workplace being detected as within range. The red dots represent GPS-defined work hours, whereas the blue dots represent GPS-defined off-work periods.

In this study, the Staff Hours app was designed to automatically record GPS data and smartphone events, including screen on or off timestamps, app notifications, and app labels. The app could track up to 5 workplace locations, and work hours recording began when the smartphone detected the workplace location within a 1-km radius for a continuous 1800-second period. Work hours recording ceased when the workplace was not detected within 1 km of the device for a continuous 1800-second period. To optimize battery usage, the GPS data were sampled at fixed intervals of 600 seconds (refer to Figure 1).

**Figure 2:**
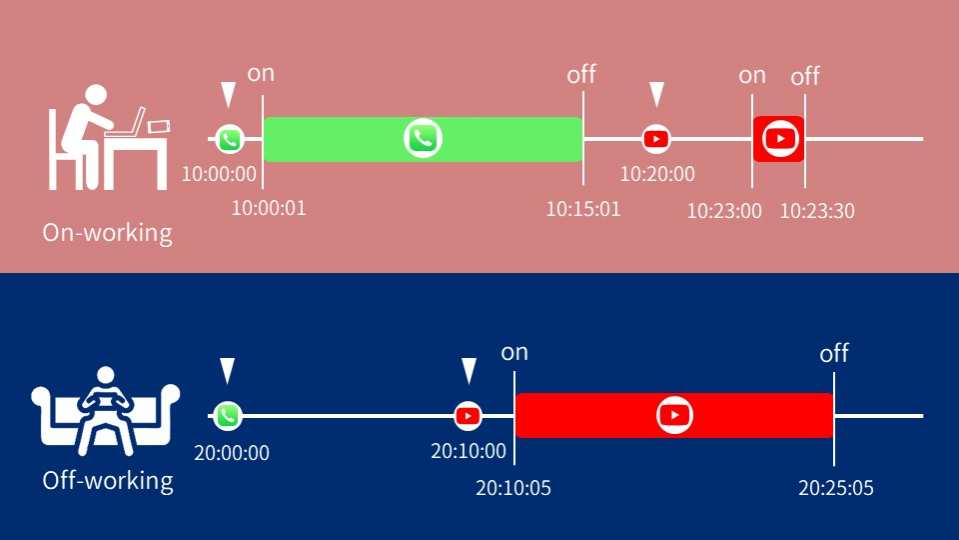
The differences in human-smartphone interaction patterns between on-working and off-working states. In this figure, a hypothetical user’s reaction time to a phone call at work was 1 second (10:00:00 to 10:00:01), and the reaction intensity was 15 minutes (10:00:01 to 10:15:01); contrastingly, there was no reaction to a phone call off work. And vice versa, the individual presented a longer reaction time and a lower reaction intensity to YouTube at work than off work. The screen events were converted into 5 features for each application (app) used within the time window of 1800 seconds. The 5 features, composed of the timestamps of screen-on or off, types of app, and notifications, were (1) the duration of app usage, (2) the frequency of notifications during each usage episode, (3) the frequency of notifications occurring outside the episode, (4) the reaction time from the moment a push notification was received to the beginning of the app usage, and (5) the reaction intensity, which was defined as the duration of the app usage following the notification.

The Android version of Staff Hours automatically captured additional smartphone events, such as timestamps of the screen on or off events, app notifications, and labels for the active apps. For example, during the off-working state between timestamps 20:00:00 and 20:30:00, the participant’s timestamps of smartphone events recorded notifications at timestamps 20:00:00 and 20:10:00. Additionally, at timestamp 20:10:05, a screen on the event was detected until timestamp 20:25:05, during which the active app label was “YouTube” (refer to Figure 2).

As the Android operating system allowed background recording of human-smartphone interaction patterns, this valuable information included app usage duration, notification frequencies during each app usage episode, occurrences of notifications outside episodes, reaction time from receiving a push notification to initiating app usage, and reaction intensity, which defined the duration of app usage following the notification.

Considering the differences in data collection capabilities between Android and iOS devices, we focused on analyzing and using human-smartphone interaction patterns specifically from the Android version of the Staff Hours app in our study. The model’s performance was assessed based on this data set, while future research could investigate whether the model’s performance differs depending on the phone operating system, considering the potential impacts of internal settings on GPS data quality, as suggested in previous research.

### Data Preprocessing

Human-smartphone interaction patterns were transformed into 5 features for each app used within an 1800-second time window. These features encompassed timestamps of the screen on or off events, app types, and notifications, quantifying app usage duration, notification frequencies during usage episodes, frequencies of notifications outside episodes, reaction time from push notification to app usage, and reaction intensity (refer to Figure 2). The total number of features quantifying human-smartphone interaction patterns was 5 times the number of apps used within each 1800-second time window. Data were excluded if no screen events were recorded on a certain day.

The machine learning algorithm was trained using GPS-defined working states, where off-working states were coded as 0 and on-working states as 1. To ensure accurate training, time windows of 1800 seconds containing both GPS-defined on-working and off-working states were excluded from the training data sets. We chose to use 1800-second time windows due to the nature of our work hours recording process, which involved detecting workplace locations for continuous 1800-second periods to commence or cease work hours recording. Additionally, we considered previous research on factors like fatigue, boredom, and smartphone use during work hours, where similar 1200-second time windows were used to capture relevant behavior [15]. Thus, we adopted time windows of 1800 seconds for our machine learning algorithm.

Data between midnight and 5:45 were excluded for several reasons: (1) this period primarily covered sleep time during a typical workday; (2) participants did not use their smartphones during sleep [6-8], which could introduce confusion between smartphone nonuse patterns during nocturnal hours and nonuse patterns during work hours in the machine learning process; and (3) the other 18.25-hour data (from 5:45 to midnight) might consist of approximately half on-working and half off-working data, given the typical work hours of 8 to 10 hours per day.

### Model Development

**Figure 3:**
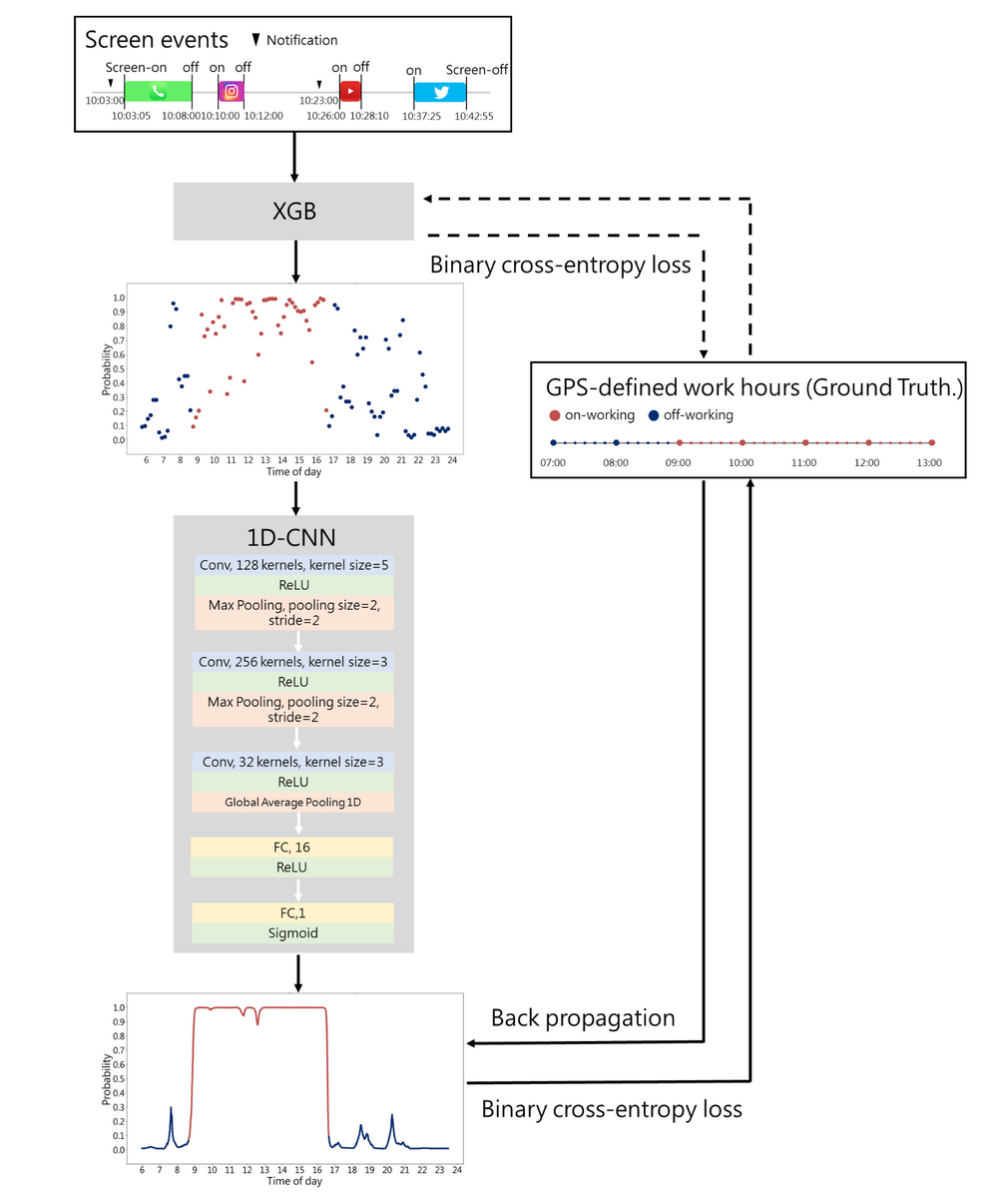
The algorithm to generate probability in work mode. Our algorithm used a 2-stage supervised machine learning process to differentiate human-smartphone interaction patterns at work and off work, which were defined by GPS locations. The first stage applied extreme gradient boosted (XGB) trees to transform human-smartphone interaction patterns into a provisional probability, and the second stage applied the 1-dimensional convolutional neural networks (1D-CNN) to yield the next-minute probabilities given the preceding probability in a temporal sequence. More specifically, the 1D-CNN model is composed of 3 convolution (Conv) layers and 2 fully connected (FC) layers. The activation function of these layers included rectified linear unit (ReLU) and sigmoid function. The first 2 convolution layers used max pooling, and the third convolution layer used global average pooling. GPS-defined on-working or off-working statuses were labeled as either 1 or 0. The trained machine learning algorithm yielded a probability between 0 and 1 from any time series of smartphone usage patterns. The higher probability in work mode generated from human-smartphone interaction patterns indicates a greater correspondence with on-working states defined by GPS. The red line represents GPS-defined work hours, whereas the blue line represents GPS-defined off-work periods.

In this study, we used a 2-stage supervised machine learning process to develop our algorithm. In the first stage, we used extreme gradient boosted trees (XGB) to transform human-smartphone interaction patterns into probabilities using softmax. In the second stage, we used the 1-dimensional convolutional neural networks (1D-CNN) model to generate successive probabilities based on preceding probabilities in a sequence (refer to Figure 3). The algorithm was trained using human-smartphone interaction patterns and the corresponding GPS-defined working status. The GPS-defined on-working or off-working statuses were labeled as either 1 or 0, resulting in a probability between 0 and 1 from any time series of human-smartphone interaction patterns (refer to Figure 3).

To address individual differences in smartphone usage, we developed separate models for each participant. We implemented machine learning algorithms with a 5-fold cross-validation approach. Missing values were imputed by sampling with replacement from nonmissing values. We used the package named TensorFlow (version 2.4.1; Google) to implement the 1D-CNN model. All data processing and analysis were performed using Python (version 3.8.5; Python Software Foundation), NumPy version 1.19.2, and scikit-learn version 0.23.2 [16].

### Statistical Analysis

#### Model Evaluation

**Figure 4:**
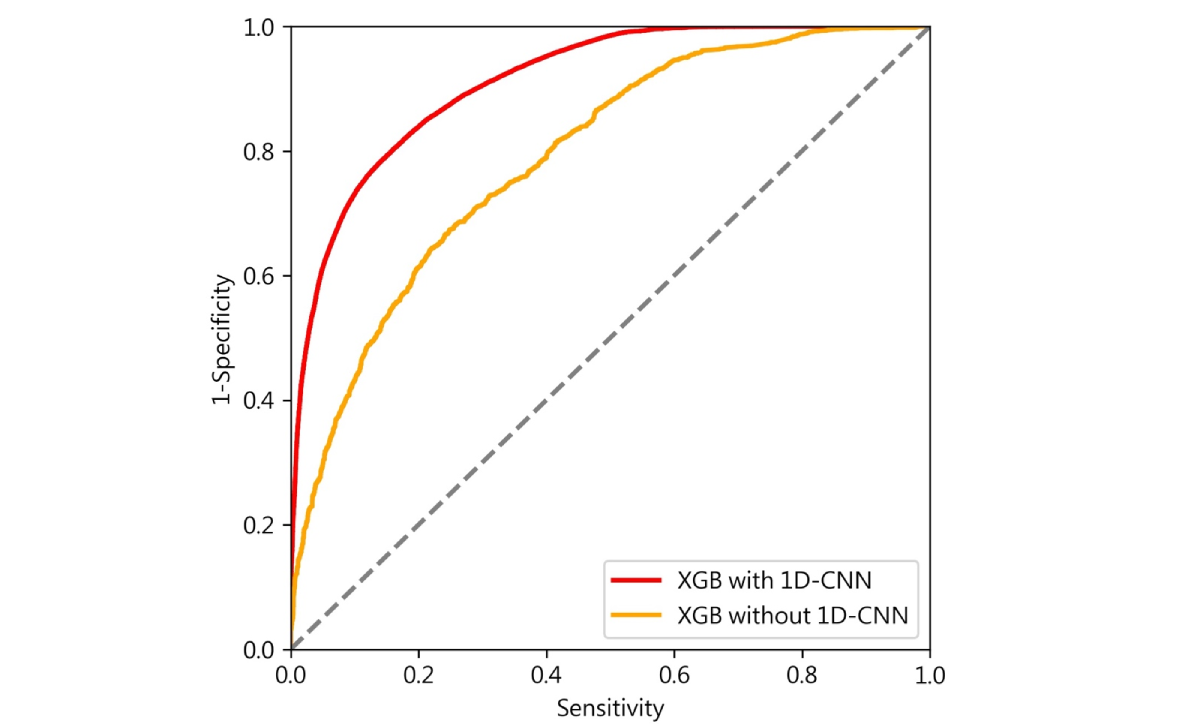
Receiver operating characteristic curve. A participant’s area under the receiver operating characteristic curve (AUC) in the first stage of this model, derived via extreme gradient boosted (XGB) trees and without 1-dimensional convolutional neural networks (1D-CNN), was 0.791, and the AUC increased to 0.912 in the final output with 1D-CNN. In this study, the average AUC derived via XGB without 1D-CNN, was 0.748 (SD 0.088), and the average AUC increased to 0.915 (SD 0.064) in the final output with 1D-CNN among 121 participants.

To evaluate the performance of our model, we measured the accuracy of each person-day and calculated the area under the receiver operating characteristic curve (AUC) for each individual. We assessed the AUC and its accuracy for days with typical work hours, holidays, and both. We excluded the transition area, defined as the 15 minutes before and after GPS data switched from on-working to off-working or vice versa, from the calculation of accuracy and the receiver operating characteristic curve analysis. On-working states were determined based on working probabilities higher than 0.5, while off-working states were defined by working probabilities lower than 0.5. These states were derived from human-smartphone interaction patterns using the machine learning algorithm. We calculated sensitivity, specificity, and overall accuracy by comparing the 2 working states estimated by the probability in work mode and GPS-defined working states. True positives were defined as on-working states identified by both the probability in work mode and GPS location, while false positives were on-working states identified solely by the probability in work mode but not by the GPS location. The algorithm’s performance was evaluated by examining each participant’s AUC [17], summarizing the model's performance across all possible thresholds and misclassification error weightings for each person-day (refer to Figure 4). We reported the overall AUC for days with typical work hours and holidays. As there were no GPS-defined work hours on holidays, we could not calculate the AUCs for holidays alone. An AUC lower than 0.7 was considered indicative of low discrimination [17].

The reference accuracy is obtained by applying a well-known “9 to 5” paradigm, which assumes that each day, regardless of being a typical workday or a holiday, has work hours from 9 AM to 5 PM, and the remaining time is considered off-working hours. Using this paradigm, we calculate the probability of correctly identifying working states for each day without prior knowledge of whether it is a workday or not. It serves as a baseline for comparison with our algorithm’s performance. It is important to note that we did not find any similar models to directly compare with ours in the recent systematic review [18]. Therefore, we used the reference accuracy method mentioned above to assess the effectiveness of our algorithm in identifying working states. We hypothesized that our algorithm, trained using data from typical work hours, would also perform effectively during holidays, accurately identifying most working probabilities lower than 0.5 based on human-smartphone interaction patterns observed during holiday periods. To evaluate our model’s performance, we conducted paired *t* tests to compare the accuracies of our algorithm with the reference accuracies for days with typical work hours, holidays, and both scenarios. This comparison provides insights into how well our algorithm performs in distinguishing working and nonworking states compared to the established “9 to 5” reference.

#### Investigation of the Minimum Number of Features

To address potential interpretability concerns related to the XGB approach, we assessed the number of smartphone usage features required in each machine learning model to achieve predictive power similar to using all features. We ranked the features for each XGB model based on their feature importance scores and identified common features with high importance scores among all participants. Using the greedy algorithm, we obtained the optimal AUC with a minimum number of variables. We set the threshold for differences between the AUC using all variables and the AUC with a minimum number of variables to be less than 0.01.

#### Investigation of the Model’s Stability

Considering the individual differences in smartphone usage, we trained separate models for each participant to achieve model robustness. To determine the number of days required in the data set for model stability, we examined the association between individuals’ AUCs and the number of days used for training. Data collected from 5 working days theoretically encompass the variety of smartphone usage patterns within a 1-week cycle.

#### Distribution of the Probabilities of Work Mode, Break Time, and Remote Work Hours

**Figure 5:**
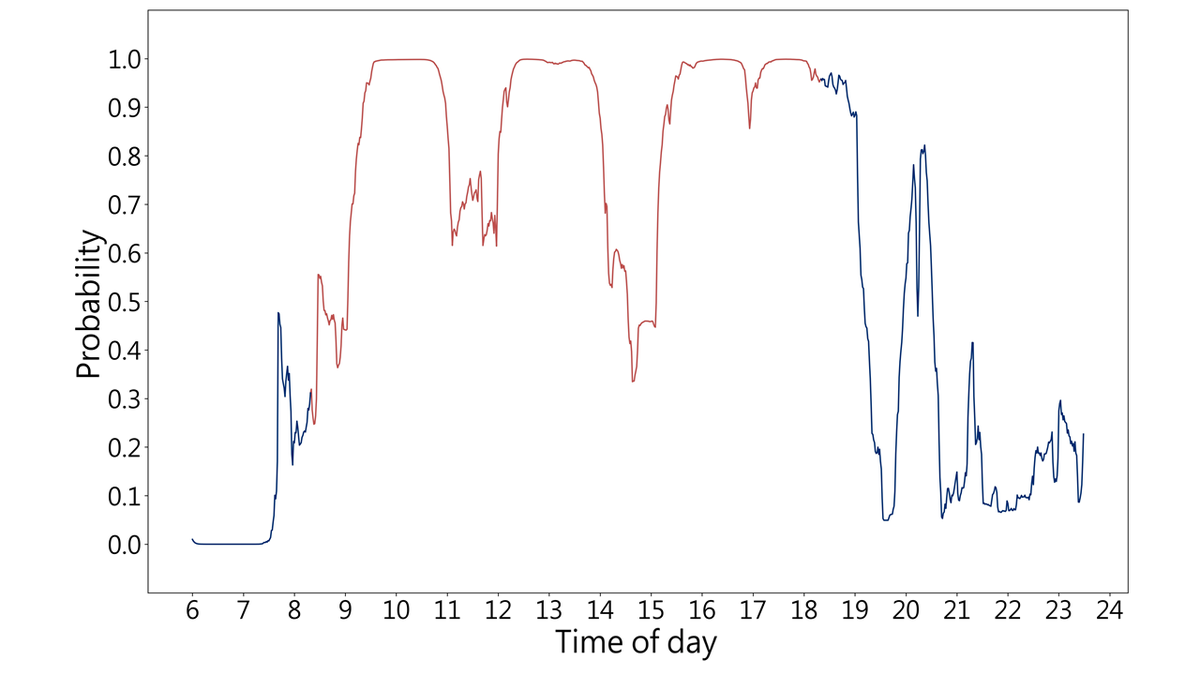
Probability in work mode diagram for 1 day. The scatter plot illustrates the proposed probability in work mode diagram for 1 day. The red line represents GPS-defined work hours, whereas the blue line represents GPS-defined off-work period. We set a threshold of 0.5 for the probability in work mode defined at work and off work. We interpreted office-working, break times, off-working, and remote working by the probability in work mode and GPS. In this scenario, the period between 9:03 to 14:32 is 1 episode of the individual’s “office working” period, during which the probability in work mode is higher than 0.5 and the GPS location is “at work.” The period between 14:33 to 15:06 is a “break time,” during which the probability in work mode falls lower than 0.5 but the GPS location is still at work. By contrast, the period between 19:58 to 20:31 is a “remote working” period, during which the probability in work mode is higher than 0.5 and the GPS location indicates “off work.”

For each person-day with typical work hours, break times were defined as periods when the probability in work mode fell below 0.5 during GPS-defined on-working states, and remote work hours were defined as periods when the probability in work mode exceeded the threshold during GPS-defined off-working states (refer to Figure 5). We compared work hours defined by human-smartphone interactions and those defined by GPS counterparts, as well as break times and remote work using paired *t* tests.

## Results

A total of 121 participants (64 women, 52.9%; mean age 37.9, SD 8.7 years), with 5503 person-days were included in this study. Most of them (64/121, 52.9%) were medical doctors. The average AUC in the first stage of this model, derived via XGB and without 1D-CNN, was 0.748 (SD 0.088), and the AUC increased to 0.915 (SD 0.064) in the final output with 1D-CNN. Among the 121 participants, 1 (0.8%) participant’s AUC was below 0.7, whereas other participants’ AUC ranged from 0.756 to 0.993. Figure 4 offers an illustrative example of the probability in a work mode diagram for 1 day, providing a visual representation of the concept.

The average accuracy of 81.9% (SD 16.0%) achieved by our algorithm was significantly higher than the reference accuracy (estimated by the “working 9 to 5” paradigm) of 73.0% (SD 16.3%) among the total 5503 person-days (*P*<.001). Among the 3762 person-days with typical work hours, the average accuracy of 83.2% (SD 12.1%) via our algorithm was significantly higher than the reference accuracy (estimated by the “working 9 to 5” paradigm) of 82.3% (SD 11.3%; *P*=.001). Additionally, for the 1741 holidays, the average accuracy of 79.1% (SD 22.0%) via our algorithm was significantly higher than the reference accuracy (estimated by “working 9 to 5”) of 53% (SD 18.5%; *P*<.001). The accuracy of typical work hours was significantly higher than that of holidays (*P*<.001).

Based on our interpretation, we calculated the following averages: (1) 9.3 (SD 2.5) hours of office working (defined by both GPS location at the worksite and probability in work mode higher than 0.5), (2) 0.9 (SD 1.2) hours of break time (GPS location at the worksite but probability in work mode lower than 0.5), (3) 4.4 (SD 2.5) hours of off-working state (defined by both GPS location off the worksite and probability in work mode lower than 0.5), and (4) 1.9 (SD 1.8) hours of remote working (GPS off the worksite but probability in work mode higher than 0.5). Figure 5 provides an illustrative example of the probability in the work mode diagram for further insights into the concept.

The average break time of 0.9 (SD 1.2) hours (54.7, SD 74.5 minutes) per day accounted for 9.2% (SD 12.5%) of GPS-defined work hours. Furthermore, the remote work time of 111.6 (SD 106.4) minutes was significantly longer than the break time of 54.7 (SD 74.5) minutes (*P*<.001). Consequently, the average work hours of 11.2 (SD 2.8) hours (669.3, SD 167.8 minutes), as defined by probability in work mode higher than 0.5, were significantly longer than the GPS-defined counterparts (612.4, SD 135.8 minutes) by 56.9 minutes (*P*<.001).

## Discussion

In this study, we developed a novel machine learning model that leveraged human-smartphone interactions and GPS location data to accurately estimate work hours, achieving an average prediction performance of 0.915. The model provided enhanced accuracy in work hour estimation, offering comprehensive insights into work patterns, including remote work and breaks. Both the accuracy on typical workdays and holidays significantly surpassed the references estimated by the “working 9 to 5” paradigm. Our approach uniquely used human-smartphone interaction patterns and GPS data collected passively through the Staff Hours app, developed by our team [4,13,14]. While the field of behavioral science has seen numerous studies using machine learning models for digital phenotyping of mental health and device use patterns [18], our study stands out as the first to explore the use of human-smartphone interaction patterns to distinguish work-related states. This innovative approach opens new possibilities for understanding work behaviors and related factors. The model’s ability to identify break times at work and remote work off-site further improved the accuracy of investigating work hours. Simultaneously recording informative features (screen events) and corresponding labels (GPS data) with high temporal resolution allowed the model to provide dense daily assessments, offering potential insights into minute-by-minute variations in work performances. Our approach was unobtrusive, imposing no additional burden on the participants beyond their normal smartphone use. Moreover, our data collection occurred in naturalistic settings, capturing human-smartphone interactions in a more ecological context than conventional assessments. This feature enhances the applicability and real-world relevance of our findings.

Our study demonstrated that machine learning and deep learning models using human-smartphone interaction patterns can effectively differentiate behaviors at work and off work. Specifically, our tree-based machine learning model (XGB) effectively classified participants’ behaviors using screen events collected passively by our app, providing an interpretable explanation through feature importance. Leveraging deep learning techniques such as 1D-CNN, we successfully decoded the probability of being in work mode in time series data. The inclusion of 1D-CNN significantly improved the AUC from 0.748 to 0.915, as it efficiently used the probabilistic information from the human-smartphone interaction feature classifier during decoding, allowing for continuous updates of the predicted probability with each new data point. Notably, the design of the combined XGB and 1D-CNN models resembled an approach used to decode words and sentences from the cerebral cortical activities of a paralyzed individual with anarthria in a previous study [19]. In that study, a neural network and a natural language model were used to decode words and sentences as the patient attempted to articulate speech. The application of the natural language model resulted in a significant improvement in word decoding accuracy from 39.5% to 74.4% [19]. This similarity in modeling strategies highlights the validity and effectiveness of individualized models, given the diverse human-smartphone interaction patterns we encountered. Moreover, while we observed substantial interindividual differences in model performance, our results revealed that 99.2% (120/121) of the participants’ AUCs reached a level with acceptable discrimination (higher than 0.7) [20,21], indicating the model’s overall effectiveness. These interindividual differences not only reflected variations in model performance but also elucidated differences in participants’ work patterns, including the proportion of break times during work hours and instances of remote work outside of the worksite.

There are several methodological limitations should be noted when interpreting our findings. First, our results assumed that the probability in work mode during “typical work hours” for medical staff in clinical practice was mostly associated with medical institutions, even during the COVID-19 outbreak. While the model demonstrated promising accuracy for medical staff, it is essential to acknowledge the limitation in generalizing our findings to other occupations. The model’s generalizability to different job contexts and settings requires further investigation. Second, a small percentage (1/121, 0.8%) of participants did not exhibit acceptable discrimination levels (AUC higher than 0.7). This could be attributed to various factors, such as individuals turning off their smartphones all day, using different smartphones for work and off-work activities, or relying on personal computers or tablets as alternatives to smartphones. In such cases, the probability in work mode failed to discriminate between on-working and off-working states. Third, the data collection efforts were limited to Android users, and we did not include data from iOS users. The Staff Hours app, which automatically recorded GPS data, was specifically designed for Android devices. As a result, we were unable to assess potential variations in the model’s predictive capabilities based on different phone operating systems. Finally, while the investigation of human-smartphone interaction behaviors is informative, it is inherently limited. However, our study contributes to the progression toward a more comprehensive, neurobehavioral-oriented investigation, providing insights into precise brain activities and their manifestation in behaviors.

In conclusion, our study presents a novel approach to predicting the probability of a smartphone user being in a work mode, using human-smartphone interaction data and machine learning techniques. The probability in work mode also exhibits the ability to identify break periods at the worksite and remote work outside of the worksite. With its basis on passively collected smartphone data with high temporal resolution, the probability in work mode can significantly enhance the precision and accuracy of work hour investigations.

## Abbreviations

1D-CNN: 1-dimensional convolutional neural network
AUC: area under the receiver operating characteristic curve
XGB: extreme gradient boosted

## Data Availability

The data sets generated and analyzed in this study are not publicly available due to the confidentiality agreement but are available from the corresponding author on reasonable request.
